# Statistics of Cases of Miasmatic Fever Treated in 1846

**Published:** 1847-04

**Authors:** 


*Statistics of Cases of Miasmatic Fever treated in* 1846. By GEORGE L. UPSHUR, M. D., of Norfolk, Virginia.

During the past year, 105 cases of miasmatic fever came under my care. Of these, 83 were Intermittent, and 22 Remittent. Of the intermittent, 1 was quartan, 15 tertian, 62 quotidian, and 5 masked. Of the masked, one took the form of neuralgia, and four simulated hysteria.

The treatment chiefly employed was the sulphate of quinine, administered in large doses, without regard to the stage of the disease. In one case—a quotidian — occurring in a youth aged 16, of sanguineous, excitable temperament, I administered 15 grains just as the cold stage was passing off. All the symptoms were ameliorated; the hot stage lasted but one hour, and the patient had no return of the disease.

In 25 cases, I gave 30 grains in five hours, during the height of the febrile stage. The pulse was *lessened in force and frequency* in every instance under this treatment, and the paroxysm cut short by the speedy appearance of perspiration. In only one of these cases was the remedy preceded by other treatment. The exception was the case of an exceedingly robust man, in whom there existed even in his ordinary health, a strong tendency of blood to the head. I bled him to twenty ounces before administering the quinine. He returned to his work (that of a baker,) forty eight hours afterwards, and had no return of the fever during the season. He told me that for several years past he had not escaped an attack of bilious fever in the autumn, and that he was usually kept in his bed for three weeks. Said that he had taken quinine before, but not while the "fever was on."

In one case, the patient was partially comatose during the first paroxysm. This condition was relieved, in a measure, by a cathartic of calomel and aloes. Three hours before the second chill was expected, I administered 25 grains quinine, and followed this by 15 grains more two hours afterwards. The patient missed the paroxysm and went to work the next day.

In 14 cases there was a recurrence of the disease. The recurrence in ten of these, however, could be positively traced to a second exposure to the causes of the affection.

The masked forms of the disease yielded readily to the quinine treatment. One of the cases which simulated hysteria was remarkably severe in its character, the patient being seized every afternoon with violent convulsions, accompanied with flushed face and considerable excitement of the circulation. She was treated at first by active purgation, and vesicants to the nucha, with only slight abatement in the intensity of the paroxysm. The regularity with which the attacks came, coupled with the fact that the patient resided in a part of the city where intermittent fever was prevailing, suggested the employment of quinine. She commenced early in the morning with five grains every hour, and took thirty grains. The paroxysm was much milder in the evening, and did not recur at all the next day. She remained well for seven days, when she was agian attacked as in the first instance, and again relieved by the same treatment. She subsequently had a third attack which was cured in the same

Her catamenia had been interrupted for six months previous to her sickness, and did not return until six weeks after the last attack.

I was not called to a single case of remittent fever at the beginning of the disease. In one case the patient had been ill *eleven* days without any treatment whatever; she was much emaciated, and had suffered from diarrhœa for six days. I gave her a table-spoonfull of the following mixture every hour:

| | | | |
|---|---|---|---|
| ℞. Quiniæ Sulph. | ʒss. | | |
| Morph. Sulph. | gr. ss. | | |
| Aquæ | f. ℥iij. | M. | |

In the course of five or six hours she perspired freely, fell into a quiet sleep, and in two days after was entirely free from disease. This was the sole treatment of the case, except the tinct. chlorid. ferri, which was given for ten days after convalescence was established.

The other cases were managed after the same manner—the large doses of quinine being preceded by a simple cathartic of jalap and bitartrate of potassa, in those cases only where there was great torpor of the bowels.

Not one of the 105 cases died, and all together did not take a drachm of calomel, or any other preparation of mercury.

I observed unpleasant symptoms in only three cases, where they seemed to be at all dependent upon the large doses of quinine.

1. A delicate nervous female, aged 36, was ordered 5 grains every hour, for a second attack of quotidian intermittent. When she had taken 20 grains, she became suddenly nauseated and vomited up three mouthfuls of scarlet blood. This occured in the morning, and the chill was expected late in the afternoon. The medicine was suspended immediately, and she missed the paroxysm, and recovered without any untoward symptom. She was treated with quinine for the first attack and also for a third, without any such effect being produced. The hæmatemesis was not vicarious of the menstrual discharge, as the catamenia had not been interupted.

2. In this case the quinine vomited the patient like full doses of tartar emetic. She took twenty grains in five grain doses in solution, combined with spt. æth. nit. There was no gastric derangement prior to the exhibition of the medicine.

3. In the third case the patient, a female aged 40, who had but recently recovered from a very severe attack of *lichen agrius*, was rendered deaf, or nearly so, for ten days, by taking forty grains of quinine in eight hours. The intermittent, a tertian, was permanently cured.

I was never deterred from giving the quinine by the existence of diarrhœa, irritability of the stomach, or headache, provided the case was urgent, and it was absolutely necessary to put an immediate stop to the paroxysms. In cases of great torpor of the bowels, if there was time to spare, I preferred to begin the treatment by purging freely, because the quinine is not readily absorbed if there is much constipation. Usually, however, the safer practice is to put an end to the paroxysms first, and afterwards attend to the local affections.

I have found great benefit from combining the sulphate of morphia with quinine, especially in those cases complicated with diarrhœa and irritable stomach. I also gave in many cases where the skin was very dry and the thirst urgent, the spt. æth. nit. combined with a solution of quinine, with great benefit.

My experience in the treatment of miasmatic fever in 1846, leads me to the following conclusions :

1. In a large majority of cases, no matter of what type the fever is, the "preparatory treatment," so called, is worse than useless, causing a loss of time which is often fatal to the patient.

2. A large dose of quinine, ( 15 or 20 grains ) administered at once, produces a more certain and permanent curative impression upon the system, than small doses ( 1 or 2 grains ) frequently repeated.

Quinine in *large* doses, when administered in the hot stage, so far from exciting the circulation, acts as a decided sedative upon it—the pulse in every instance lessening in force and frequency under its influence. The dogma, therefore, that "quinine in fever is poison," must be discarded.

4. In *uncomplicated* miasmatic fever, mercurials are not at all essential to a complete and permanent cure. They may sometimes be given with advantage in cases where cathartics are indicated at the onset of the disease.—Medical Examiner.

*Norfolk, Va., February* 6, 1847.

